# In this issue

**DOI:** 10.1111/cas.14964

**Published:** 2022-11-03

## 1 Abnormality of apico–basal polarity in adenocarcinoma


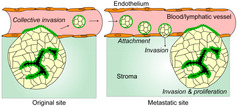


Epithelial cells line the surfaces of organs and blood vessels in our body to form the epithelium, a layer which is crucial for biological functions such as absorption and secretion of key molecules, as well as protection.

In order to function properly, epithelial cells need to maintain their polarity. What exactly does this mean? When these cells line ducts in our bodies, they are positioned so that their “apical” side is towards the hollow part of the duct (its lumen), and their “basal” part is away from it, towards the basement membrane. Establishing and maintaining this polarity involves a plethora of molecular interactions, and a cascade of cell signaling pathways. Any disturbance in epithelial polarity could leading to significant functional changes. For example, loss of epithelial cell polarity due to dysregulation of polarity‐determinant proteins is believed to play a key role in cancer progression.

In this review, the authors have highlighted the current knowledge of polarity changes in differentiated cancers like colorectal carcinomas, whose cells resemble normal cells more closely than other cancer cells, in terms of structure and organization.

Recent advancements in 3D cell culture have contributed to our understanding of cancer progression. 3D culture studies of human colorectal cancer have revealed that the polarity of these cells switches rapidly when surrounding conditions are changed. It turns out that this polarity switching is also linked to cancer metastasis.

Authors also point out that invasive micropapillary carcinoma (MPC), a type of cancer in which cell polarity is reversed, offers an important experimental system. By probing Madin‐Darby canine kidney (MDCK) cells, which is a classical model of polarity, researchers have found one of the possible mediators of polarity reversal in cancerous cells: abnormal RHO–RHO‐associated kinase (ROCK) signaling.

Further, researchers are trying to understand the functional implications of reversed cell polarity in differentiated cancers. Currently, there are no particular treatments targeting this reversal of polarity. With more studies throwing light on the mechanistic and functional aspects of polarity changes, though, novel treatment approaches may emerge.https://onlinelibrary.wiley.com/doi/full/10.1111/CAS.15549

## 2 Identification and validation of a poor clinical outcome subtype of primary prostate cancer with midkine abundance


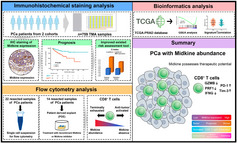


Prostate cancer (PCa) is one of the most prevalent types of cancer, which causes significant mortality among men. Localized PCa is usually treated via a radical prostatectomy procedure, for the removal of the prostate gland. Unfortunately, many patients experience biochemical recurrence (BCR) or PCa relapse after this procedure, which is associated with an elevated risk of mortality. Hence, it is crucial to identify molecular biomarkers that can predict the likelihood of BCR, and to improve the accuracy of currently used predictors.

Midkine (MDK) is a heparin‐binding, multifunctional protein which plays a key role in the progression of cancer. In particular, it is known to enhance the immunosuppressive microenvironment which induces tumor growth in melanoma. It also compromises the anti‐tumor activity of naïve CD8^+^ T cells, which in turn enhances tumor growth in glioma. However, it is unclear if MDK modulates immune suppression in PCa.

This study aimed to understand the role of MDK in the progression of PCa. A retrospective analysis of 759 patients with PCa who underwent radical prostatectomy with available tumor microarrays was performed to assess the immune infiltration and clinical relevance. The study also included 325 patient records from The Cancer Genome Atlas database as an external cohort, to analyze their mRNA data and clinical information. In addition, 22 fresh tumor samples were selected for flow cytometry analyses, and 14 samples for cell culture analyses.

The findings revealed a strong correlation between MDK expression in PCa tumor cells and tumor progression. In addition, the study found that incorporating MDK expression into a risk assessment tool known as CAPRA‐S increased its accuracy in predicting BCR.

The study also explored the connection between MDK expression and the clinical outcome of tumor infiltrating CD8+ T cells. Findings indicated that PCa tumors that expressed high levels of MDK reduced the cytolytic ability (ability to destroy cells) of CD8^+^ T cells. However, this did not affect the tumor infiltrating capacity of these cells.

This indicates that MDK has a direct immunosuppressive effect on CD8^+^ T cells but does not function as a chemoattractant. Interestingly, the inhibition of MDK led to the reactivation of CD8^+^ T cells' anti‐tumor activity. Importantly, tumors with MDK abundance were resistant to hormonal therapy but sensitive to post‐operative radiotherapy. To sum up, intratumoral MDK expression can act as an effective and independent predictor of BCR in postoperative patients with PCa. These findings could be of great significance in advancing the role of MDK as a therapeutic target for PCa.

https://onlinelibrary.wiley.com/doi/full/10.1111/CAS.15546

## 3 Profiling chromosomal‐level variations in gastric malignancies


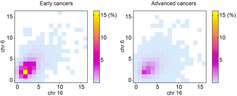


Aneuploidy, a condition where a cell contains abnormal number of chromosomes due to improper segregation, is known to be a distinct characteristic of cancer cells. Several studies have tried to explain the correlation between aneuploidy and tumor formation but have, unfortunately, been met with the “aneuploidy paradox,” characterized by contradictions between experimental models and clinical cancers. A thorough evaluation of patient samples at the chromosomal level is, therefore, necessary for a better understanding.

To this end, Negoto et al., in a new study, assessed the ploidy (the number of chromosomes present in a cell) of gastric cancer cells ranging from early to advanced stages using a technique called “fluorescence *in situ* hybridization” (FISH), which uses a fluorescent tagged probe (a small DNA fragment) to locate a specific DNA sequence on a set of chromosomes. They analyzed 190 samples from patients with gastric cancer (63 early cancer and 127 advanced cancer cases) to investigate the impact of chromosomal aberrations on the invasiveness of cancer.

The FISH analysis revealed that advanced stage cancer cells had an increased and more varied level of aneuploidy compared to those at early stages. Interestingly, aneuploidy increased significantly as the cancer cells penetrated deeper into the layers of the stomach. This suggested that aneuploidy may have a role to play in tumor invasion and metastasis (spread of cancer cells to different parts of the body). A comparison of aneuploidy among different parts within a tumor as well as between primary cancers and metastasized tumors showed that the extent of aneuploidy is quite constant in primary cancers. This could, however, change with metastasis, as was revealed by the existence of both increased and decreased aneuploidy in metastatic tumors.

In addition to aneuploid cells, a significant amount of polyploid cells (cells having double or triple the standard number of chromosomes) were also found in affected stomach lining. Further exploration into the multiplication of aneuploid cells revealed a close association with p53, a tumor suppressor protein that prevents tumor formation by stopping cells containing damaged DNA from dividing. Any changes to p53 or its absence could, therefore, lead to uncontrolled cell division, i.e., cancer. Accordingly, the authors found that p53 deficiency was directly linked to a marked increase in aneuploidy and its expansion.

These findings shed light on the involvement of chromosomal alterations in gastric cancers and could pave the way for further research on understanding ploidy and its role in the malignant potential of tumors.

https://onlinelibrary.wiley.com/doi/full/10.1111/cas.15544

